# Developing the HLS_19_-YP12 for measuring health literacy in young people: a latent trait analysis using Rasch modelling and confirmatory factor analysis

**DOI:** 10.1186/s12913-022-08831-4

**Published:** 2022-12-06

**Authors:** Christopher Le, Øystein Guttersrud, Kristine Sørensen, Hanne Søberg Finbråten

**Affiliations:** 1 Department of Health and Nursing Sciences, Faculty of Social and Health Sciences, Inland Norway University of Applied Sciences, PO Box 400, N-2418 Elverum, Norway; 2 Norwegian Centre for Science Education, Faculty of Mathematics and Natural Sciences, University of Oslo, Blindern, PO Box 1106, N-0317 Oslo, Norway; 3 Global Health Literacy Academy, Viengevej 100, D-8240 Risskov, Denmark

**Keywords:** Confirmatory factor analysis of categorical data, Health literacy measurement, HLS_19_-YP12, Rasch modelling, Short version, Young people

## Abstract

**Background:**

Accurate and precise measures of health literacy (HL) is supportive for health policy making, tailoring health service design, and ensuring equitable access to health services. According to research, valid and reliable unidimensional HL measurement instruments explicitly targeted at young people (YP) are scarce. Thus, this study aims at assessing the psychometric properties of existing unidimensional instruments and developing an HL instrument suitable for YP aged 16–25 years.

**Methods:**

Applying the HLS_19_-Q47 in computer-assisted telephone interviews, we collected data in a representative sample comprising 890 YP aged 16–25 years in Norway. Applying the partial credit parameterization of the unidimensional Rasch model for polytomous data (PCM) and confirmatory factor analysis (CFA) with categorical variables, we evaluated the psychometric properties of the short versions of the HLS_19_-Q47; HLS_19_-Q12, HLS_19_-SF12, and HLS_19_-Q12-NO. A new 12-item short version for measuring HL in YP, HLS_19_-YP12, is suggested.

**Results:**

The HLS_19_-Q12 did not display sufficient fit to the PCM, and the HLS_19_-SF12 was not sufficiently unidimensional. Relative to the PCM, some items in the HLS_19_-Q12, the HLS_19_-SF12, and the HLS_19_-Q12-NO discriminated poorly between participants at high and at low locations on the underlying latent trait. We observed disordered response categories for some items in the HLS_19_-Q12 and the HLS_19_-SF12. A few items in the HLS_19_-Q12, the HLS_19_-SF12, and the HLS_19_-Q12-NO displayed either uniform or non-uniform differential item functioning. Applying one-factorial CFA, none of the aforementioned short versions achieved exact fit in terms of non-significant model chi-square statistic, or approximate fit in terms of SRMR ≤ .080 and all entries ≤ .10 that were observed in the respective residual matrix. The newly suggested parsimonious 12-item scale, HLS_19_-YP12, displayed sufficiently fit to the PCM and achieved approximate fit using one-factorial CFA.

**Conclusions:**

Compared to other parsimonious 12-item short versions of HLS_19_-Q47, the HLS_19_-YP12 has superior psychometric properties and unconditionally proved its unidimensionality. The HLS_19_-YP12 offers an efficient and much-needed screening tool for use among YP, which is likely a useful application in processes towards the development and evaluation of health policy and public health work, as well as for use in clinical settings.

**Supplementary Information:**

The online version contains supplementary material available at 10.1186/s12913-022-08831-4.

## Background

In several Western countries, young people (YP) from the age of 16 are expected to take responsibility for health on their own [1]. Today, YP are frequently exposed to health-related information from different sources, such as peers, adults, social media, and commercial enterprises [2]. Several studies have shown that YP might lack sufficient health literacy (HL) to access, understand, critically appraise, and use such information [3, 4].

YP from the age of 16 report worse access to healthcare than does the adult population [1]. According to Levesque et al.’s conceptualization of access to healthcare [5], there are five corresponding abilities of the populations required to generate access: ability to perceive, ability to seek, ability to reach, ability to pay, and ability to engage. These required abilities reflect the importance of individuals’ HL in different health-related situations, e.g., accessing the health services.

Sufficient HL might empower YP to deal with health information, enable, and access health-promoting activities [6]. According to the HLS-EU Consortium, “Health literacy is linked to literacy and entails people’s knowledge, motivation and competences to access, understand, appraise, and apply health information in order to make judgments and take decisions in everyday life concerning healthcare, disease prevention, and health promotion to maintain or improve quality of life during the life course” [7]. Based on the comprehensive definition, the HLS-EU Consortium [7] developed a conceptual model and an associated framework for questionnaire item development, which combined three health domains (HDs) and four cognitive domains (CDs) operationalized into a 12-cell matrix. Subsequently, the 12-cell matrix focuses on finding (F), understanding (U), judging (appraising; J), and applying (A) health information concerning healthcare (HC), disease prevention (DP), and health promotion (HP).

Accurate and precise measurement is vital for identifying vulnerable groups with low HL that might need support in managing health issues, suggesting tailored interventions, and evaluating progress in HL promotion [8]. Only when population HL is appropriately described, the public health and health care services can make targeted prioritizations, become more efficient, continuously improve the quality of services towards vulnerable groups, and contribute to increasing population HL [9]. During the past decades, more than 200 tools have been developed focusing on various aspects of HL [10]. The inconsistencies due to instrument diversity have complicated the interpretation of findings across studies, as well as the choice of instruments for new studies [11, 12]. Another major challenge is that different instruments and tools measure different aspects of HL owing to different definitions, contexts, and/or subpopulations [13].

Several reviews of measurement instruments for youth HL have been published to date [14–17]. The systematic review of generic HL measurement instruments for children and adolescents [15] revealed that most instruments did not provide sufficient conceptual information, as they only measured the researchers’ own contextual understanding of HL. A more recent systematic review [18] also uncovered an inconsistency in how researchers define HL versus develop measures of HL, in which there is a high risk of missing information necessary to understand the underlying conceptualization of HL in the studies. Subsequently, Guo et al. [14] suggested that most studies on the use of HL instruments applied to children and adolescents were of poor methodological quality, and involved vague descriptions of the target population. Moreover, the best-developed HL instrument for young people (HLAT-8) identified in their review has not been tested for adolescents under 18. The instrument is multidimensional, and was not conceptually developed based on a theoretical framework.

The European Health Literacy Survey Questionnaire (HLS-EU-Q) is widely used to measure HL in adult populations. It was developed on basis of the 12-cell conceptual model of Sørensen et al. [7], reflecting people’s proficiency in finding, understanding, appraising, and applying health information across three health domains: HC, DP, and HP. Several short versions of this comprehensive instrument have been suggested (see the Table 1). As opposed to the 12-item short versions, the 16-item short version, HLS-EU-Q16, does not reflect the 12-cell matrix. The present study, therefore, excluded the 16-item version from the comparative analyses of the short versions. In 2019, the WHO Action Network on Measuring Population and Organisational Health Literacy (M-POHL) revised the HLS-EU-Q47 items for the HLS_19_ instrument in terms of rewording items and adding/removing instruction details, such as examples within items [19]. Furthermore, the HLS_19_ Consortium also suggested an additional 12-item short version: HLS_19_-Q12. The revised HLS_19_-Q47 and the short version HLS_19_-Q12 were applied in the HLS_19_ survey to measure general HL in the adult population in 17 countries. The Table 1 below provides an overview of the HLS_19_ instrument and its short versions.

**Table 1:** Overview of HLS-EU/HLS_19_-Q47 and suggested short versions

Original	Developed by:	HLS_19_ version	Revised by:
HLS-EU-Q47	HLS-EU Consortium (2012) [20]	HLS_19_-Q47	HLS_19_ Consortium (2021) [19]
HLS-EU-Q16	HLS-EU Consortium (2012) [20]	HLS_19_-Q16	HLS_19_ Consortium (2021) [19]
**Short version**	**Suggested by:**	**HLS**_**19**_** version**	**Validated by:**
HLS-Q12	Finbråten et al. (2018) [21]	HLS_19_-Q12-NO	Le et al. (2021) [22]
HLS-SF12	Duong et al. (2019) [23]	HLS_19_-SF12	Present study
		**HLS**_**19**_** version**	**Suggested by:**
		HLS_19_-Q12	HLS_19_ Consortium (2021) [19]
		HLS_19_-YP12	Present study

The psychometric properties of the HLS-EU-Q47 have been widely assessed using several techniques, such as principal component analysis (PCA) [24, 25], confirmatory factor analysis (CFA) [26–29], and Rasch modelling [21, 23, 30]. Also, the short versions of HLS-EU-Q47; HLS-EU-Q16 [20], HLS-Q12 [21], HLS-SF12 [23], and HLS_19_-Q12 [19], have been suggested [19–21, 23] and validated for adult populations [31, 32], but not in YP. Nonetheless, Okan et al. [15] concluded that there still is a lack of valid and reliable unidimensional scales for measuring general HL explicitly targeted at YP.

Consequently, our aims are to: (1) evaluate the psychometric properties of the 12-item short versions of the HLS_19_-Q47 in YP and (2) consecutively suggest a parsimonious unidimensional short version suitable for measuring general HL among YP. Specifically, the hypothesis is that when applied in YP aged 16–25, the short versions of the HLS_19_-Q47 achieve approximate fit and display acceptable goodness of fit-indices when evaluated using CFA, and are sufficiently unidimensional, well-targeted scales with acceptable person separation (reliability), consisting of independent and invariant items at the ordinal level (i.e., ordered response categories) each displaying sufficient fit to the unidimensional Rasch model. This hypothesis forms the basis for comparison against the psychometric properties of the consecutively suggested parsimonious unidimensional short version: HLS_19_-YP12.

## Methods

### Sampling and data collection

This study used data from the Norwegian part of the Health Literacy Survey 2019–2021 (HLS_19_) [22], which was collected during April–October 2020. The Norwegian HLS_19_ study applied a population-based cross-sectional survey study design, and was funded by the Norwegian Directorate of Health. The survey was conducted in cooperation with Oslo Metropolitan University and Inland Norway University of Applied Sciences. A Norwegian market research agency (Norstat), with access to country representative strata, collected the data using computer-assisted telephone interviewing (CATI). The data collection was performed in two steps. In the first step (*n* = 3000) data on the comprehensive 47-item instrument were collected, whereas in the second step (*n* = 3000) data were collected only on the two short versions: HLS_19_-Q12-NO and HLS_19_-Q12. Out of 6000 participants, 890 participants met our inclusion criteria “YP aged 16–25 years”, and 419 responded to the comprehensive scale HLS_19_-Q47.

#### Characteristics of the participants

The study’s sample included 890 participants with a slight predominance of males (Table 2). Due to the stepwise data collection, only the smaller sample (n = 419) was applicable to the scales: HLS_19_-YP12, HLS_19_-SF12, and HLS_19_-Q47. Most of the participants have an education equal to upper secondary school or lower. Two-thirds report belonging to the upper social level, and above three quarters report no economic deprivation. Most of the participants also report being healthy.

**Table 2:** Distribution of participants’ sociodemographic factors

Characteristic	n (%), *n* = 890^a^	n (%), *n* = 419^b^
**Age**		
16-20yo	436 (49.0)	230 (54.9)
21-25yo	454 (51.0)	189 (45.1)
**Gender**		
Male	459 (51.6)	209 (49.9)
**Education**		
Below and equal to upper secondary school	684 (76.9)	324 (77.3)
Above upper secondary school	201 (22.6)	93 (22.2)
Missing	5 (0.6)	2 (0.5)
**Economic deprivation**		
Yes	90 (10.1)	43 (10.3)
No	704 (79.1)	334 (79.7)
Missing	96 (10.8)	42 (10.0)
**Social status**		
1–5	245 (27.5)	117 (27.9)
6–10	591 (66.4)	273 (65.2)
Missing	54 (6.1)	29 (6.9)
**Long-term illness**		
Yes	204 (22.9)	99 (23.6)
No	682 (76.6)	318 (75.9)
Missing	4 (0.4)	2 (0.5)
**Health status**		
Mostly healthy	777 (87.3)	370 (88.3)
Increased risk or having chronic health problem	106 (11.9)	47 (11.2)
Missing	7 (0.8)	2 (0.5)

^a^ Applicable to only HLS_19_-Q12, and HLS_19_-Q12-NO

^b^Applicable to HLS_19_-YP12, HLS_19_-Q12, HLS_19_-SF12, HLS_19_-Q12-NO, HLS_19_-Q47

### Measures, translation, and cultural adaptations

In combination with the HL scales, we collected person factors and covariates, such as age, gender, education, self-reported level in the society, economic deprivation, long-term illness, and health status. In addition, the HL-scales have been culturally adapted and translated into Norwegian as described below.

#### The HLS_19_-Q47 and its 12-item short versions

The HLS_19_-Q47 and its 12-item short versions (see the Table 1) reflect the conceptual model of Sørensen et al. [25], and uses a 4-point rating scale with the response categories: (1) very difficult, (2) difficult, (3) easy, and (4) very easy. Moreover, the “don’t know” response category was used when stated spontaneously by the participants, which was recoded to missing data in the analyses.

#### Translation and cultural adaptation of the HLS_19_-Q47

The translation of the HLS_19_-Q47 was performed in accordance with Brislin’s protocol [33]. The questionnaire was translated from English to Norwegian by two bilingual persons (translators) independently. The concept of HL was deeply understood by the translators, and they were experienced questionnaire developers. The two translators compared their translated versions and discussed item content and wording. A third person read the Norwegian translation, made comments, and suggested amendments. A professional translator was engaged to do a back-translation when consensus had been reached. The original English version was then compared with the back-translated version, in order to gain the most semantically, technically, and contextually equivalent versions. Finally, the translation was quality-assured by the data collection agency (Norstat). To ensure that the item contents were understood and could be considered relevant also in a Norwegian context, cognitive interviews with a think aloud-procedure [34] were conducted when translating the HLS-EU-Q47 [30]. The results from these cognitive interviews were monitored as part of the translation process in the current study.

#### Pilot testing of the instruments

Prior to the main data collection, a pilot of the instruments was conducted in several institutions and organizations, such as municipalities, directorates, universities, NGOs, and hospitals. Some HLS_19_-Q47 items were revised based on results from the pilot survey. These amendments were based on empirical observations interpreted in light of theoretical expectations.

### Model estimation

#### Rasch modelling

There are three main item response theory (IRT) models: 1) the one-parameter IRT model, 2) the two-parameter model, and 3) the three-parameter model. The one-parameter IRT model corresponds to the Rasch model. Distinct from other IRT models, the Rasch models meet requirements of fundamental measurement, such as sufficiency [35], additivity [36], invariance [37], and specific objectivity [38]. On this background, the unidimensional Rasch model was applied in this study.

We tested data up against the partial credit parameterization [39] of the unidimensional Rasch model for polytomous data [40], and up against the partial credit parameterization of the “between-item” “multidimensional random coefficients multinomial logit” (MRCML) model [41]. The latter was used when testing the HLS_19_-Q47 data up against a 12-dimensional model that reflects all 12 cells in the HLS-EU HL matrix: three health domains by four cognitive domains (12 correlated sub-scales). Using the unidimensional approach, we assume perfectly correlated subscales, that is, three perfectly aligned health domains (HP, DP, and HC) and/or four perfectly aligned cognitive domains (F, U, J and A). Using the three- and 12- dimensional approaches, we relax this constraint and allow health domains and/or cognitive domains to covary. Additionally, consecutive approach (treating the subscales as orthogonal or uncorrelated) was used when assessing item invariance. Models were estimated by applying the ConQuest 5 software [42] and the RUMM2030plus software [43].

For item-location estimates, RUMM2030plus uses pairwise maximum likelihood estimation (PMLE) [44], while ConQuest 5 uses marginal maximum likelihood estimation (MMLE) [45]. Normality may be considered a prerequisite when using maximum likelihood estimation. As such, the raw data obtained from the scales measuring YP’s HL were transformed into person-location estimates (logit values) using RUMM2030plus and ConQuest 5 software. Subsequently, the transformed data could be considered continuous and at interval level, and there is evidence of data normality when examining the normal distribution histograms. For unbiased person-location estimates, both softwares apply Warm’s mean weighted likelihood estimation (WLE) [46]. The average item-location estimate was set to 0.0 in all analyses.

Using Rasch measurement theory, we evaluated dimensionality, response dependency, targeting, reliability, item fit, differential item functioning (DIF), and ordering of response categories.

##### Dimensionality

For each of the instrument versions, the dimensionality was assessed applying the combined principal component analysis (PCA) of residuals and paired *t*-test procedure [43, 47]. Based on the PCA, two subsets of items were identified. Person-location estimates on the respective two subsets were then compared using paired *t*-test. Multidimensionality is indicated when the proportion of individuals with significantly different person-location estimates on the compared subscales exceeds 5% [47, 48]. Unidimensionality is deemed to be strictly proved as opposed to multidimensionality [49]. Given a normal distribution of the differences in person-location estimates derived from the two subsets, Tennant and Pallant [50] claimed that this approach is robust enough to detect multidimensionality. In such a case, where the proportion of individuals with significantly different person-location estimates on the compared subscales exceeds 5%, we also manually performed the binomial test, which is an exact test of the statistical significance of deviations from a theoretically expected distribution of observations into two categories. If the proportion lower bound 95% confidence interval in terms of number of significant t-tests is lower than or equal to 0.05 (5%), then the scale could be considered sufficiently unidimensional.

##### Response dependency

Effective instruments do not collect redundant information and are free from response dependency, which is present when responses to an item are statistically dependent on the responses to a previous item. The average of the residual correlations added to 0.2 (average + 0.2) was used as a cut-off to indicate possible “significant” response dependency [51]. When the responses to a pair of items are locally dependent, we may construct a subtest or, when developing instruments, delete one of the items.

##### Targeting of persons and items

For a well-targeted scale, the distribution of the person-estimates should match the distribution of the item threshold estimates or difficulties [52]. As the scale is always centered on zero logits in the Rasch software, the mean person location value for a well-targeted scale would be close to the value of zero. Poor targeting may result in deflated variance in person estimates, which subsequently leads to poor person separation and deflated “test–retest” reliability indexes.

##### Reliability – internal consistency

The person separation reliability (PSR) and the person separation index (PSI) were estimated using the ConQuest 5 software and the RUMM2030plus software, respectively. In addition, Omega was estimated using the Mplus 8.6 software and the Microsoft excel-based tool to calculate ordinal Omega by standardized factor loadings and standardized residual variances [53]. Frisbie [54] has suggested that the reliability of the sum scores should exceed 0.85 or 0.65 when drawing conclusions at the individual or group level, respectively.

##### Individual item fit

Using ConQuest 5, weighted Mean Square Error (infit MNSQ) or variance-weighted fit residual was used to indicate individual item fit to the Rasch model [55]. The expected infit MNSQ value is 1, which implies perfect data-model fit. Using instruments at the population level, we consider 0.7 > infit < 1.3 as sufficient [32, 56]. Furthermore, item under- and over-discrimination relative to Rasch models was indicated by values significantly different from the expected value of 1 with an absolute value of the T statistic higher than 1.96 [55, 57]. Under-discriminating items most likely measure too much of “something else” that does not correlate positively with the latent trait, with the result that they will not discriminate sufficiently well between persons with high and low standing on the latent trait [58].

A non-significant chi-square item fit statistic (*p* > 0.05) indicates good data-model fit, but the probability of detecting significant values or “misfit items” increases by the number of significance tests performed. The Bonferroni correction is one of several methods to counteract this effect [59]. For a 12-item scale, the Bonferroni adjusted chi-square probability is p/12 = 0.05/12 = 0.004.

##### Differential item functioning

A central requirement of the Rasch model is measurement invariance, which means that items should function in the same way across different groups of people [60], such as gender and people with different health status. Items display differential item functioning (DIF) when items have different relative difficulty (uniform DIF) or discriminate differently (non-uniform DIF) for different groups of people.

We explored whether the items displayed DIF for selected person factors by two-way analysis of variance (ANOVA) of standardized residuals and inspecting graphical displays [60]. Owing to the inclusion criteria “YP aged 16–25 years”, we dichotomized participants’ highest education level (“upper secondary school or below” versus “above upper secondary school”), and we dichotomized participants’ age accordingly (16–20 years old versus 21–25 years old). Participants’ self-reported social status on a scale from 1 to 10 was dichotomized, as the two age groups probably define their level in the society based on different criteria due to life experiences: education level, living conditions, and economic status. Economic deprivation was present, as some reported difficulties with paying bills at the end of the month. Participants described their health status (mostly healthy or increased risk of/having a chronic health problem) and reported whether they suffered from long-term illness expected to last or had lasted for at least six months.

##### Ordered response categories

Polytomous items (here: 4-point response scale) with ordered response categories yield categorical data at the ordinal level. This implies significantly different and ordered thresholds, where thresholds are the locations at the latent trait where adjacent response categories are equally likely [60]. Disordered thresholds indicate response categories not working as intended [61].

#### Confirmatory factor modelling

Using the software Mplus 8.6 [62], one- and three-factorial CFAs of the HLS_19_-YP12, HLS_19_-Q12, HLS_19_-SF12, and HLS_19_-Q12-NO data, were conducted to examine the correlation structure and item loadings in light of the theoretical framework – the HLS-EU health literacy matrix [7]. The one-, two-, three-, four- and 12-factorial CFAs of the HLS_19_-Q47 data were supplementarily performed to assist confirmation of prior studies.

Following Asparouhov and Muthén [63], a significant model chi-square statistic implies that the suggested confirmatory factor model fails the “exact fit test”. Applying categorical data, weighted least square (WLS) estimator was used to obtain the model chi-square statistic [64]. Other fit indices were estimated using robust diagonally weighted least squares (WLSMV) estimator: a default option for categorical data in Mplus 8.6. Using WLSMV estimators with ordered-category data, polychoric correlation coefficients were estimated and reported in Table 3.

**Table 3:** Descriptive statistics and correlation matrix of HLS_19_-YP12, with variances on the diagonal

Item no. and in HLS_19_-Q47	On a scale from very easy to very difficult, how easy would you say it is to	1	2	3	4	5	6	7	8	9	10	11	12
1: HL04	…find out where to get professional help when you are ill? [Instructions: such as doctor, nurse, pharmacist, psychologist]	1.000	.353	.302	.409	.260	.282	.306	.294	.257	.252	.253	.354
2: HL07	…understand information about what to do in a medical emergency?	.353	1.000	.381	.383	.377	.360	.451	.359	.359	.321	.303	.363
3: HL10	…judge the advantages and disadvantages of different treatment options?	.302	.381	1.000	.474	.303	.358	.417	.259	.437	.377	.362	.312
4: HL13	…use information your doctor gives to you to make decisions about your illness?	.409	.383	.474	1.000	.313	.374	.409	.342	.378	.360	.333	.402
5: HL18	…find information on how to handle mental health problems? [Instructions: stress, depression, or anxiety]	.260	.377	.303	.313	1.000	.322	.334	.262	.286	.292	.281	.304
6: HL23	…understand information about recommended health screenings or examinations? [Instructions: e.g., colorectal cancer screening, measuring blood pressure, blood sugar test]	.282	.360	.358	.374	.322	1.000	.427	.375	.327	.406	.361	.325
7: HL26	…judge which vaccinations, you or your family may need?	.306	.451	.417	.409	.334	.427	1.000	.436	.340	.274	.326	.360
8: HL30	…decide how you can protect yourself from illness using advice from family or friends?	.294	.359	.259	.342	.262	.375	.436	1.000	.319	.286	.253	.266
9: HL36	…find information about how to promote health at work, at school or in the neighborhood?	.257	.359	.437	.378	.286	.327	.340	.319	1.000	.338	.428	.446
10: HL38	…understand information on food packaging?	.252	.321	.377	.360	.292	.406	.274	.286	.338	1.000	.190	.316
11: HL41	…judge how your neighborhood may affect your health and well-being? [Instructions: Your community, your neighborhood]	.253	.303	.362	.333	.281	.361	.326	.253	.428	.190	1.000	.334
12: HL46	…influence your living conditions that affect your health and well-being? [Instructions: Drinking and eating habits, exercise etc.]	.354	.363	.312	.402	.304	.325	.360	.266	.446	.316	.334	1.000
N		406	399	393	403	407	406	403	409	401	411	398	405
Percentage (%) distribution of “very difficult” responses		2	3	3	1	6	1	2	2	2	2	6	2
Percentage (%) distribution of “difficult” responses		12	23	40	19	36	15	31	24	32	20	40	17
Percentage (%) distribution of “easy” responses		42	53	46	59	43	54	46	54	52	47	42	59
Percentage (%) distribution of “very easy” responses		44	21	12	21	15	30	21	20	14	30	11	22

Using WLSMV estimators with ordered-category data in Mplus 8.6, polychoric correlation coefficients were reported in Table 3

Other absolute fit indices below their target value, such as the standardized root mean square residual (SRMR ≤ 0.080) combined with small residual correlation matrix entries [63] (i.e., absolute value ≤ 0.10) [65], indicate approximate fit. Other “goodness of fit” (GOF) indices (with target value in parenthesis) may assist model evaluation, such as the root mean square error of approximation (RMSEA ≤ 0.06), comparative fit index (CFI ≥ 0.95), and Tucker-Lewis index (TLI ≥ 0.95) [66]. However, RMSEA values ≤ 0.08 may be considered acceptable in a small sample, whereas the other GOF indices suggest a good model fit. Additionally, CFI between 0.90 and 0.95 also indicates reasonable fit, while values < 0.90 are considered poor fit [67].

#### Developing the HLS_19_-YP12

The suggested 12-item short version in the present study was developed from analyses of the HLS_19_-Q47 and the other three 12-item short versions, applied in YP aged 16–25 in Norway. The development was stepwise: 1) exclude items that in the Rasch analyses displayed poor fit, DIF, disordered response categories, and that might collect redundant information; and 2) using CFA to assess the fit statistics, in which large residual correlation matrix entries indicate the need for model modifications. Items included in the suggested version were continuously ensured reflecting the conceptual 12-cell matrix.

#### Handling missing data

Missing data also comprises “don’t know” responses, which on average made up 2 percent of the data. The highest missing rates (5–7%) were observed for items 2, 3, 10, 11, 19 and 34, while items 8, 14, 22, 32, 33, 37, 38, 39, 40, 42, 43 and 44 had less than 1% missing values. However, using full information maximum likelihood (FIML) estimation, person-locations and item-thresholds are estimated based on available information [62].

## Results

### Descriptive statistics and correlations between the items of HLS_19_-YP12

For all items, the percentage of participants who had the “difficult” and “very difficult” responses is lower than the percentage for responses of “easy” and “very easy” (Table 3). The most difficult items were item41, item10, and item18 with 46, 43, and 42% of (very) difficult responses, respectively. The easiest items were item4, item23, item46, and item13 with 86, 84, 81, and 80% of (very) easy responses, respectively. The correlations between the items of HLS_19_-YP12 could be considered small to medium (range: 0.190 – 0.474).

### Overall data-model fit and unidimensionality of 12-item short versions

The HLS_19_-YP12, the HLS_19_-Q12-NO, and the HLS_19_-SF12 data displayed sufficiently overall fit to the PCM (non-significant overall chi-square statistic), while the HLS_19_-Q12 data did not. All short versions explored in our study had reliability indexes (PSR, PSI and Omega) above 0.65. The HLS_19_-YP12, the HLS_19_-Q12, and the HLS_19_-Q12-NO are considered sufficiently unidimensional, while the HLS_19_-SF12 is not (Table 4).

**Table 4:** Overall data-model fit, reliability, and unidimensionality by applying Rasch modelling of the 12-item short scales

	HLS_19_-YP12	HLS_19_-Q12	HLS_19_-SF12	HLS_19_-Q12-NO
	Present study	HLS_19_ Consortium (2021) [19]	Duong et al. (2019) [23]	Finbråten et al. (2018) [21]
**Unidimensionality t-tests (CI)**^**RUMM**^				
Number significant tests	16	27	34	17
Out of:	415	413	414	414
Dim(%)	3.86%	6.54%	8.21%	4.11%
Proportion lower 95% CI	1.8%	4.4%	6.1%	2.0%
**Chi-square interaction**^**RUMM**^				
Total item chi-square	49.54	72.11	56.72	61.17
df	48	48	48	48
Probability	0.41	0.01 ^a^	0.18	0.10
**Mean (SD) in logits**^**RUMM**^				
Item fit residual	0.12 (1.01)	-0.01 (1.08)	0.17 (1.07)	0.04 (1.08)
Person fit residual	-0.40 (1.46)	-0.41 (1.40)	-0.36 (1.31)	-0.36 (1.34)
**Mean person location in logits**^**RUMM**^	1.035	1.155	1.141	1.084
**Reliability**				
Omega (by Excel-based tool)^Mplus^	0.86	0.85	0.84	0.84
PSI based on PMLE^RUMM^	0.82	0.81	0.81	0.79
PSR (MMLE/WLE)^CQ^	0.829/0.827	0.816/0.815	0.812/0.809	0.809/0.808
**Log-likelihoods**^**CQ**^				
Deviance (ep)	9,659 (37)	9,666 (37)	9,679 (37)	9,772 (37)
AIC (ep)	9,733 (37)	9,740 (37)	9,753 (37)	9,846 (37)

*df* Degree of freedom, *SD* Standard deviation, *SE* Standard error, *Omega* Internal consistency reliability, *PSI* Person separation index, *PSR* Person separation reliability, *PMLE* Pairwise maximum likelihood estimate, *MMLE* Marginal maximum likelihood estimate, *WLE* Warm's mean likelihood estimate, *Deviance* Deviance statistics, *ep* Total number of estimated parameters, *AIC* Akaike Information Criterion, *RUMM* RUMM2030 software, *CQ/ConQuest* ConQuest 5 software, *Mplus* Version 8.6

^a^ total item chi-square is significant at 5%-level indicating significant deviation between the observed data and what was expected from the Rasch model; dim(%): proportion of individuals with significantly different person-location estimates (below 5% confirms unidimensionality); proportion lower 95% CI: lower than 5% confirms acceptable unidimensionality

No response dependency was observed for any short version, but the HLS_19_-Q47 suffers from serious local dependency with up to 35 pairs of dependent items when applying the unidimensional PCM. For details, see Supplementary Table S1.

**Fig. 1:**
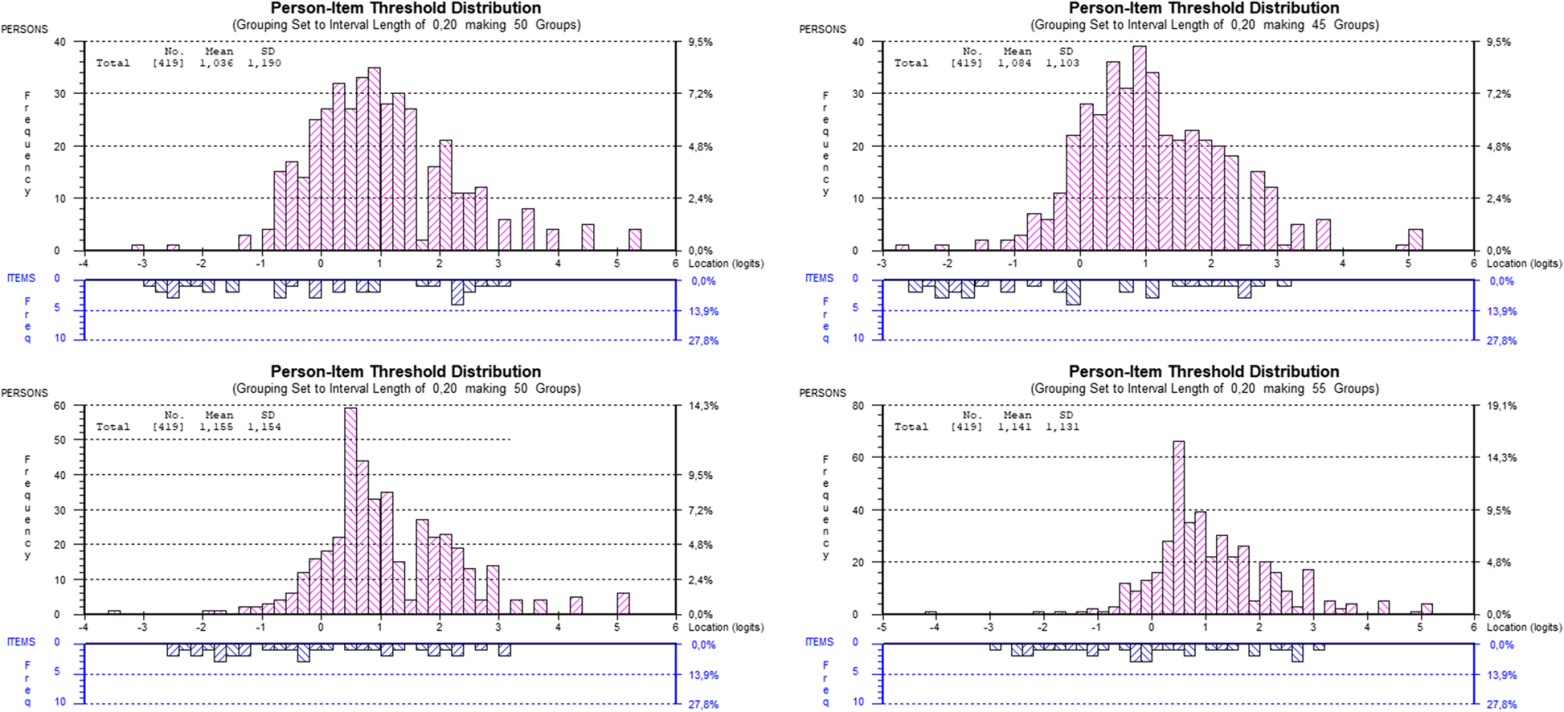
Targeting, person-item threshold distribution of 12-item short versions. *Note*: Targeting of HLS_19_-YP12, HLS_19_-Q12, HLS_19_-SF12, and HLS_19_-Q12-NO reflects the person location mean in Table 4 indicating a slight right-skewness given the item location mean calibrated to be at 0.0 logits

No short version was particularly well-targeted to the YP, but the distribution of item-threshold locations and the distribution of person locations were best aligned for the HLS_19_-YP12 (Fig. 1); mean person location for the scales HLS_19_-YP12, HLS_19_-Q12, HLS_19_-SF12, and HLS_19_-Q12-NO were 1.035, 1.155, 1.141, and 1.084, respectively (Table 4).

#### Exploring dimensionality by using confirmatory factor analysis

Comparing one- and three-factorial models, only the one-factor model of HLS_19_-YP12 achieved approximate fit with acceptable SRMR (0.030) and with no entry in the residual correlation matrix > 0.10 (Table 5). Supplementary Table S2 provides an overview of all entries in the residual correlation matrix based on all four 12-item scales, applying both one- and three-factor models. Other GOF indices indicated that the model-implied correlation matrix sufficiently well re-created the observed correlation matrix: RMSEA (0.039; 0.034), CFI/TLI (0.985/0.981; 0.989/0.986) (Table 5). Results related to the comprehensive scale HLS_19_-Q47 are supplementarily reported in Supplementary Table S3.

**Table 5:** Fit statistics for different factor structures of 12-item short versions applying CFA

**Model**	**Short version**	${{\varvec{\chi}}}_{{\varvec{M}}}^{2}\boldsymbol{ }({\varvec{d}}{\varvec{f}}),\boldsymbol{ }{\varvec{p}}$	**SRMR**	${\varvec{N}}{\varvec{o}}.{{\varvec{r}}}_{{\varvec{r}}{\varvec{e}}{\varvec{s}}}$ $(>.10)$	**RMSEA (CI)**	**CFI/TLI**
one-factor	HLS_19_-YP12	135.48 (54), .000	.030	none	.039 (.024-.053)	.985/.981
	HLS_19_-Q12	174.13 (54), .000	.039	6 (-.18 – .14)	.057 (.044-.070)	.963/.955
	HLS_19_-SF12	211.74 (54), .000	.052	9 (-.17 – .17)	.078 (.066-.090)	.926/.909
	HLS_19_-Q12-NO	174.42 (54), .000	.042	5 (-.20 – .14)	.061 (.048-.074)	.958/.948
three-factor: HC,DP,HP	HLS_19_-YP12	115.20 (51), .000	.028	1 (-.13)	.034 (.015-.049)	.989/.986
	HLS_19_-Q12	163.64 (51), .000	.037	3 (-.15 – .12)	.054 (.040-.067)	.969/.959
	HLS_19_-SF12	188.16 (51), .000	.047	10 (-.14 – .13)	.072 (.060-.085)	.940/.922
	HLS_19_-Q12-NO	164.96 (51), .000	.039	5 (-.18 – .12)	.057 (.044-.070)	.965/.955

${\chi }_{M}^{2}$: model chi-square, called either minimum fit function chi-square or likelihood ratio chi-square, was estimated using WLS estimator. If the fit of an over-identified model SRMR (Standardized Root Mean Square Residual or standardized difference between observed and model-implied data): values ≤ .050 is good and ≤ .080 is sufficient. SRMR is used as index for approximate fit if model Chi-square is significant; $No.{r}_{res}$: number of residuals with a value > .10; (> .10) = highest value > .10; RMSEA (Root Mean Square Error of Approximation): values ≤ .06 indicate good model fit; CFI (Comparative Fit Index) and TLI (Tucker-Lewis index): values ≥ .95 are generally used as an indicator of acceptable model fit

*HC* Health Care, *DP* Disease Prevention, *HP* Health Promotion

While all short versions: HLS_19_-YP12, HLS_19_-Q12, HLS_19_-SF12, and HLS_19_-Q12-NO, achieved SRMR < 0.080 for both one- and three-factorial models, the HLS_19_-SF12 had most entries in the residual correlation matrix > 0.10, whereas the HLS_19_-YP12 had none for the one-factor model and only one high entry (-0.13) for the three-factor model. Among the 12-item short scales, the HLS_19_-YP12 obtained the most acceptable standardized factor loadings applying the one-factor structure model (all items > 0.500) (Table 6).

**Table 6:** Factor loadings for the items in the respective 12-item short versions when a one-factor structure model is considered

HLS_19_-YP12		HLS_19_-Q12		HLS_19_-SF12		HLS_19_-Q12-NO	
Present study		HLS_19_ Consortium (2021) [19]		Duong et al. (2019) [23]		Finbråten et al. (2018) [21]	
**Item no**	**F1**	**Item no**	**F1**	**Item no**	**F1**	**Item no**	**F1**
COREHL4	.513	COREHL4	.572	COREHL2	**.429**	COREHL2	**.394**
COREHL7	.627	COREHL7	.594	COREHL6	.505	COREHL7	.573
COREHL10	.630	COREHL10	.536	COREHL10	.518	COREHL10	.544
COREHL13	.658	COREHL16	.645	COREHL15	.566	COREHL14	.597
COREHL18	.513	COREHL18	.532	COREHL18	.525	COREHL18	.566
COREHL23	.613	COREHL23	.617	COREHL23	.637	COREHL23	.616
COREHL26	.648	COREHL24	.569	COREHL26	.618	COREHL28	**.430**
COREHL30	.538	COREHL31	**.425**	COREHL30	.576	COREHL30	.532
COREHL36	.620	COREHL32	.562	COREHL33	.519	COREHL32	.591
COREHL38	.535	COREHL37	.622	COREHL39	.588	COREHL38	.605
COREHL41	.542	COREHL42	.550	COREHL43	.601	COREHL43	.618
COREHL46	.588	COREHL44	.602	COREHL45	.534	COREHL44	.582

### Rasch analyses at item level for HLS_19_-YP12, HLS_19_-Q12, HLS_19_-SF12, and HLS_19_-Q12-NO

#### Individual item fit

Applying unidimensional Rasch modelling, all items for all short versions had acceptable infit values (Tables 7, 8, 9 and 10). For the HLS_19_-Q12, item31 had a T-value of 2.1 meaning that the item under-discriminated relative to the PCM. In addition, Bonferroni-adjusted chi-square probability (chi-square: 21.18; *p* < 0.001) for item42 in the same scale was significant (not reported in the Tables). Significant total item chi-square (Table 4) indicated also problems at the individual item level. Following this problem, Class Interval main effect indicating item misfit was also observed for this item concerning all person factor variables: age, gender, education, economic deprivation, level in society, long-term illness, and health status. Class Interval main effect was also observed, but only for the person factor “long-term illness”, in item45 in the HLS_19_-SF12 scale. Supplementary investigation of the HLS_19_-Q47 showed, however, there were five items (29, 34, 38, 41, 45) in the 12-dimensional model that under-discriminated relative to the PCM (Supplementary Table S4).

**Table 7:** Item characteristics, ordering of response categories, and DIF of the 12-item short version HLS_19_-YP12

**HD**	**CD**	**Item no**	**RW in HLS**_**19**_	**Item:On a scale from very difficult to very easy, how easy would you say it is:**	**1-dimensional analysis HLS**_**19**_**- YP12**							
					**ConQuest**				**RUMM**			
					**Infit**^**w**^** MNSQ**	**CI**		**T- value**	**Item estimate**	***SE***	**Ordered**	**DIF**^**a**^
						**lb**	**ub**					
HC	F	4	x	…to find out where to get professional help when you are ill?	1.07	0.86	1.14	1.0	-0.525	0.077	yes	none
	U	7	x	…to understand information about what to do in a medical emergency?	0.99	0.86	1.14	-0.1	0.101	0.078	yes	none
	J	10		…to judge the advantages and disadvantages of different treatment options?	0.98	0.87	1.13	-0.3	0.422	0.084	yes	none
	A	13	x	…to use information your doctor gives to you to make decisions about your illness?	0.93	0.86	1.14	-1.0	-0.283	0.087	yes	none
DP	F	18	x	…to find information on how to handle mental health problems?	1.09	0.87	1.13	1.3	0.537	0.074	yes	none
	U	23	x	…to understand information about recommended health screenings or examinations?	0.92	0.86	1.14	-1.2	-0.481	0.083	yes	none
	J	26	x	…to judge which vaccinations, you or your family may need?	0.97	0.87	1.13	-0.5	-0.058	0.079	yes	none
	A	30	x	…to decide how you can protect yourself from illness using advice from family or friends?	1.04	0.87	1.13	0.5	-0.089	0.081	yes	none
HP	F	36	x	…to find information about how to promote health at work, at school or in the neighborhood?	0.97	0.87	1.13	-0.4	0.057	0.084	yes	none
	U	38		…to understand information on food packaging?	1.02	0.87	1.13	0.4	-0.224	0.076	yes	none
	J	41	x	…to judge how your neighborhood may affect your health and well-being?	1.10	0.87	1.13	1.4	0.775	0.077	yes	none
	A	46	x	…to influence your living conditions that affect your health and well-being?	0.99	0.86	1.14	-0.1	-0.233	0.085	yes	none

*LS* Level in the society, *LT* Long-term illness, *H* Health status, *HD* Health domains, *CD* Cognitive domains, *HC* Health care, *DP* Disease prevention, *HP* Health promotion, *RW* Rewording, *F* Find, *U* Understand, *J* Judge, *A* Apply, *lb* Lower bound, *ub* Upper bound, *CI* Confidence interval, *T-value* Similar to the z standardized fit statistics in unidimensional Rasch analyses, *MNSQ* Mean square value, *SE* Standard Error

^w^ weighted fit MNSQ, unidimensional model using ConQuest 5

^u^ A t-value > 1.96 indicates a poorly fitting item in terms of under-discrimination relative to the Rasch model

DIF: differential item functioning; ^a^Bonferroni-adjusted 5% has been used to assist detecting possible significant deviations due to DIF; ^b^ uniform DIF; ^c^ non-uniform DIF; ^g^ Graphical only; Full-CI: Class Interval main effect applied to age, gender, education, economic deprivation, level in society, long-term illness and health status; ^nb^ Full-CI significant at 5%-level, but not significant when adjusted for Bonferroni 5%-level; Ordered: "no" refers to an item with disordered response categories

**Table 8:** Item characteristics, ordering of response categories, and DIF of the 12-item short version HLS_19_-Q12

**HD**	**CD**	**Item no**	**RW in HLS**_**19**_	**Item:On a scale from very difficult to very easy, how easy would you say it is:**	**1-dimensional analysis HLS**_**19**_**- Q12**							
					**ConQuest**				**RUMM**			
					**Infit**^**w**^** MNSQ**	**CI**		**T- value**	**Item estimate**	***SE***	**Ordered**	**DIF**^**a**^
						**lb**	**ub**					
HC	F	4	x	…to find out where to get professional help when you are ill?	1.00	0.86	1.14	0.1	-0.410	0.077	yes	**LS**^g,b^
	U	7	x	…to understand information about what to do in a medical emergency?	0.99	0.86	1.14	-0.1	0.239	0.078	yes	none
	J	10		…to judge the advantages and disadvantages of different treatment options?	1.07	0.87	1.13	1.1	0.542	0.083	yes	none
	A	16	x	…to act on advice from your doctor or pharmacist?	0.93	0.84	1.16	-0.9	-0.542	0.086	**no**	none
DP	F	18	x	…to find information on how to handle mental health problems?	1.06	0.87	1.13	0.9	0.676	0.074	yes	none
	U	23	x	…to understand information about recommended health screenings or examinations?	0.93	0.86	1.14	-1.0	-0.334	0.083	yes	none
	J	24	x	…to judge if information on unhealthy habits, such as smoking, low physical activity or drinking too much alcohol, are reliable?	0.99	0.87	1.13	-0.1	-0.231	0.078	yes	none
	A	31	x	…to decide how you can protect yourself from illness using information from the mass media?	^u^1.14	0.87	1.13	^u^2.1	0.693	0.079	yes	Full-CI^nb^
HP	F	32	x	…to find information on healthy lifestyles such as physical exercise, healthy food, or nutrition?	1.02	0.87	1.13	0.3	-0.405	0.074	yes	none
	U	37	x	…to understand advice concerning your health from family or friends?	0.95	0.85	1.15	-0.7	-0.347	0.089	yes	none
	J	42	x	…to judge how your housing conditions may affect your health and well-being?	1.08	0.86	1.14	1.2	0.084	0.075	yes	Full-CI
	A	44	x	…to make decisions to improve your health and well-being?	1.00	0.87	1.13	0.0	0.035	0.077	yes	none

*LS* Level in the society, *LT* Long-term illness, *H* Health status, *HD* Health domains, *CD* Cognitive domains, *HC* Health care, *DP* Disease prevention, *HP* Health promotion, *RW* Rewording, *F* Find, *U* Understand, *J* Judge, *A* Apply, *lb* Lower bound, *ub* Upper bound, *CI* Confidence interval, *T-value* Similar to the z standardized fit statistics in unidimensional Rasch analyses, *MNSQ* Mean square value, *SE* Standard Error

^w^ weighted fit MNSQ, unidimensional model using ConQuest 5

^u^ A t-value > 1.96 indicates a poorly fitting item in terms of under-discrimination relative to the Rasch model

DIF: differential item functioning; ^a^Bonferroni-adjusted 5% has been used to assist detecting possible significant deviations due to DIF; ^b^ uniform DIF; ^c^ non-uniform DIF; ^g^ Graphical only; Full-CI: Class Interval main effect applied to age, gender, education, economic deprivation, level in society, long-term illness and health status; ^nb^ Full-CI significant at 5%-level, but not significant when adjusted for Bonferroni 5%-level; Ordered: "no" refers to an item with disordered response categories

**Table 9:** Item characteristics, ordering of response categories, and DIF of the 12-item short version HLS_19_-SF12

**HD**	**CD**	**Item no**	**RW in HLS**_**19**_	**Item:On a scale from very difficult to very easy, how easy would you say it is:**	**1-dimensional analysis HLS**_**19**_**-SF12**							
					**ConQuest**				**RUMM**			
					**Infit**^**w**^** MNSQ**	**CI**		**T- value**	**Item estimate**	***SE***	**Ordered**	**DIF**^**a**^
						**lb**	**ub**					
HC	F	2		…to find information on treatments of illnesses that concern you?	1.06	0.86	1.14	0.8	0.214	0.083	yes	none
	U	6	x	…to understand the instruction leaflets that come with your medicine?	1.06	0.87	1.13	0.9	0.042	0.075	yes	**H**^g,b^
	J	10		…to judge the advantages and disadvantages of different treatment options?	1.03	0.87	1.13	0.5	0.527	0.083	yes	none
	A	15	x	…to call an ambulance in a medical emergency?	0.98	0.82	1.18	-0.2	-0.425	0.077	**no**	none
DP	F	18	x	…to find information on how to handle mental health problems?	1.04	0.87	1.13	0.7	0.673	0.073	yes	none
	U	23	x	…to understand information about recommended health screenings or examinations?	0.94	0.86	1.14	-0.9	-0.352	0.083	yes	none
	J	26	x	…to judge which vaccinations, you or your family may need?	0.99	0.88	1.12	-0.2	0.097	0.078	yes	none
	A	30	x	…to decide how you can protect yourself from illness using advice from family or friends?	1.00	0.87	1.13	0.0	0.065	0.081	yes	none
HP	F	33	x	…to find information about activities that are good for your mental health and well-being?	1.08	0.88	1.12	1.3	-0.587	0.077	yes	none
	U	39	x	…to understand information in the mass media on how to improve your health?	0.98	0.87	1.13	-0.2	0.212	0.081	yes	none
	J	43	x	…to judge which everyday habits affect your health?	0.95	0.86	1.14	-0.7	-0.482	0.083	yes	none
	A	45	x	…to join a sports club or exercise group if you want to be physically active?	1.08	0.87	1.13	1.1	0.015	0.068	yes	**LT**^c^

*LS* Level in the society, *LT* Long-term illness, *H* Health status, *HD* Health domains, *CD* Cognitive domains, *HC* Health care, *DP* Disease prevention, *HP* Health promotion, *RW* Rewording, *F* Find, *U* Understand, *J* Judge, *A* Apply, *lb* Lower bound, *ub* Upper bound, *CI* Confidence interval, *T-value* Similar to the z standardized fit statistics in unidimensional Rasch analyses, *MNSQ* Mean square value, *SE* Standard Error

^w^ weighted fit MNSQ, unidimensional model using ConQuest 5

^u^ A t-value > 1.96 indicates a poorly fitting item in terms of under-discrimination relative to the Rasch model

DIF: differential item functioning; ^a^Bonferroni-adjusted 5% has been used to assist detecting possible significant deviations due to DIF; ^b^ uniform DIF; ^c^ non-uniform DIF; ^g^ Graphical only; Full-CI: Class Interval main effect applied to age, gender, education, economic deprivation, level in society, long-term illness and health status; ^nb^ Full-CI significant at 5%-level, but not significant when adjusted for Bonferroni 5%-level; Ordered: "no" refers to an item with disordered response categories

**Table 10:** Item characteristics, ordering of response categories, and DIF of the 12-item short version HLS_19_-Q12-NO

**HD**	**CD**	**Item no**	**RW in HLS**_**19**_	**Item:On a scale from very difficult to very easy, how easy would you say it is:**	**1-dimensional analysis HLS**_**19**_**- Q12-NO**							
					**ConQuest**				**RUMM**			
					**Infit**^**w**^** MNSQ**	**CI**		**T- value**	**Item estimate**	***SE***	**Ordered**	**DIF**^**a**^
						**lb**	**ub**					
HC	F	2		…to find information on treatments of illnesses that concern you?	1.10	0.86	1.14	1.3	0.173	0.082	yes	none
	U	7	x	…to understand information about what to do in a medical emergency?	1.00	0.86	1.14	0.0	0.187	0.077	yes	none
	J	10		…to judge the advantages and disadvantages of different treatment options?	1.02	0.87	1.13	0.3	0.501	0.082	yes	none
	A	14		…to follow instructions on medication?	0.98	0.86	1.14	-0.2	-0.668	0.082	yes	**LS**^**b**^
DP	F	18	x	…to find information on how to handle mental health problems?	1.01	0.87	1.13	0.1	0.619	0.073	yes	none
	U	23	x	…to understand information about recommended health screenings or examinations?	0.94	0.86	1.14	-0.9	-0.390	0.082	yes	none
	J	28	x	…to judge if the information on health risks in the mass media is reliable?	1.13	0.87	1.13	1.9	0.660	0.075	yes	none
	A	30	x	…to decide how you can protect yourself from illness using advice from family or friends?	1.03	0.87	1.13	0.5	0.003	0.081	yes	none
HP	F	32	x	…to find information on healthy lifestyles such as physical exercise, healthy food, or nutrition?	1.00	0.87	1.13	0.0	-0.449	0.074	yes	none
	U	38		…to understand information on food packaging?	0.96	0.87	1.13	-0.6	-0.128	0.075	yes	none
	J	43	x	…to judge which everyday habits affect your health?	0.94	0.86	1.14	-0.9	-0.500	0.082	yes	none
	A	44	x	…to make decisions to improve your health and well-being?	1.00	0.87	1.13	0.0	-0.008	0.076	yes	none

*LS* Level in the society, *LT* Long-term illness, *H* Health status, *HD* Health domains, *CD* Cognitive domains, *HC* Health care, *DP* Disease prevention, *HP* Health promotion, *RW* Rewording, *F* Find, *U* Understand, *J* Judge, *A* Apply, *lb* Lower bound, *ub* Upper bound, *CI* Confidence interval, *T-value* Similar to the z standardized fit statistics in unidimensional Rasch analyses, *MNSQ* Mean square value, *SE* Standard Error

^w^ weighted fit MNSQ, unidimensional model using ConQuest 5

^u^ A t-value > 1.96 indicates a poorly fitting item in terms of under-discrimination relative to the Rasch model

DIF: differential item functioning; ^a^Bonferroni-adjusted 5% has been used to assist detecting possible significant deviations due to DIF; ^b^ uniform DIF; ^c^ non-uniform DIF; ^g^ Graphical only; Full-CI: Class Interval main effect applied to age, gender, education, economic deprivation, level in society, long-term illness and health status; ^nb^ Full-CI significant at 5%-level, but not significant when adjusted for Bonferroni 5%-level; Ordered: "no" refers to an item with disordered response categories

#### Differential item functioning—DIF

**Fig. 2:**
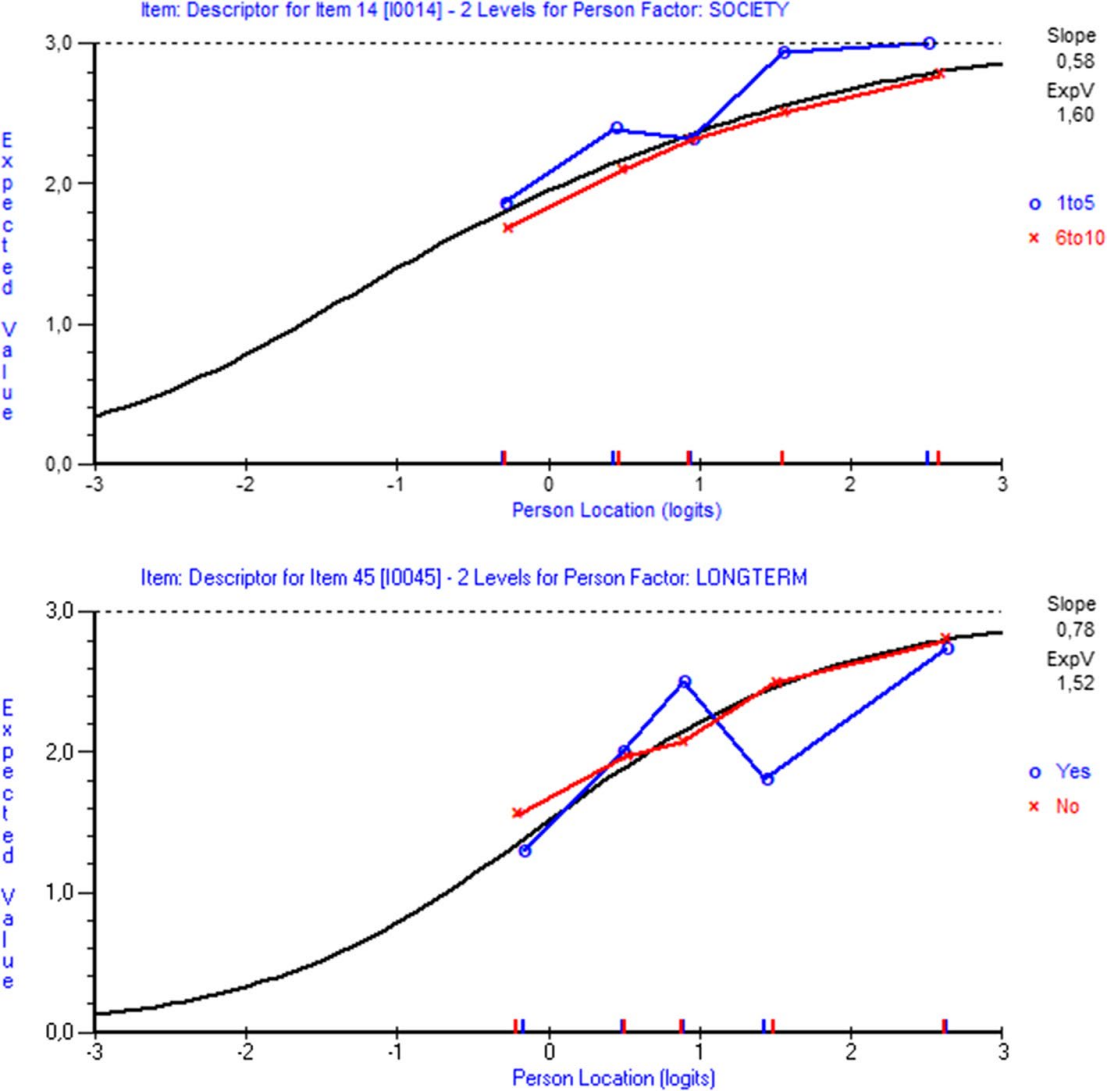
Items displaying differential item functioning – DIF. *Note*: DIF with parallel slopes is referred to as uniform DIF, whereas non-uniform DIF is present when locations are the same but the slopes are different or both the locations and slopes are different

While there was no DIF observed, neither graphical nor by significant ANOVA tests, for any item in the HLS_19_-YP12, significantly uniform DIF was observed for the HLS_19_-Q12-NO in item14 for the “level in society” subgroups, whereas item45 in the HLS_19_-SF12 scale displayed significantly non-uniform DIF for the “long-term illness” subgroups (Fig. 2). Disregarding statistical Bonferroni-adjusted non-significance, investigation of the items using the item characteristic curves (ICCs) graphically displayed uniform DIF for the HLS_19_-Q12 in item42 for the “level in society” subgroups and for the HLS_19_-SF12 in item6 for the “health status” subgroups (not reported in the Figures).

#### Ordering of response categories

**Fig. 3:**
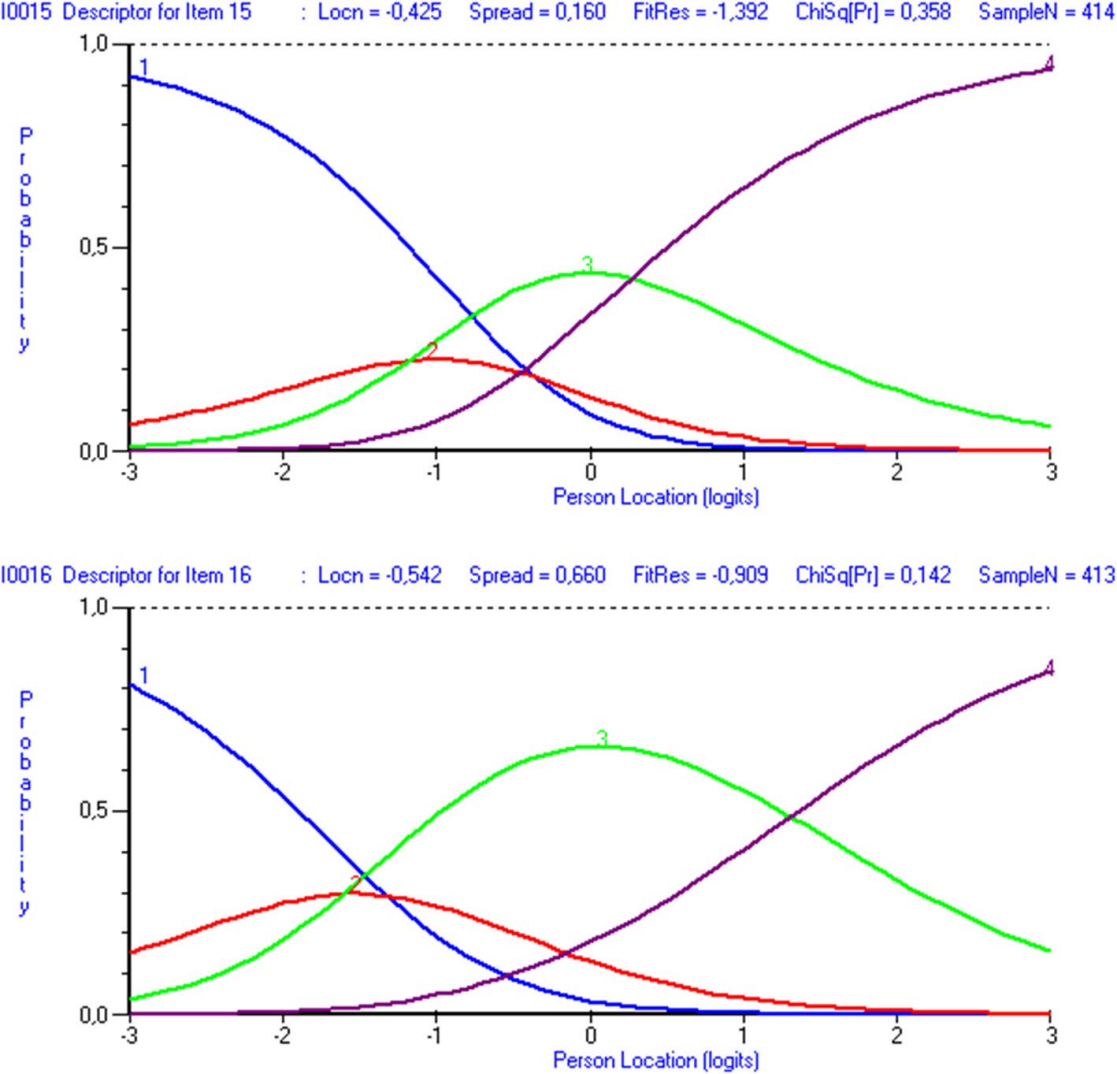
Visualization of disordered response categories of item15 and item16 in the HLS_19_-SF12 and the HLS_19_-Q12, respectively. *Note*: Using RUMM2030plus software, we observed that the category probability curves for item15 in the HLS_19_-SF12 and item16 in the HLS_19_-Q12 indicated disordered/reversed response categories. The response category 2 in both items was not the most likely for any location on the latent trait scale and might weaken the hypothesis of ordinal data. Disordered response categories were also observed for item21 in the HLS_19_-Q47 applying unidimensional Rasch model

Among the four short versions, only item15 in the HLS_19_-SF12 and item16 in the HLS_19_-Q12 displayed disordered response categories. Figure 3 shows that response category “2” in both items was not the most likely category for any location on the continuum of person location estimates.

## Discussion

In several Western health care systems, the patient role has been redefined expecting patients to be a more active part in his/her care and decision-making [68]. Accurate and precise measure of HL is very supportive for tailoring the communication between patients and health providers during the patient pathway. Similarly for the targeted public health measures. All this also applied to YP from the age of 16.

Despite the fact that the HLS_19_-Q47 and its short versions, HLS_19_-Q12, HLS_19_-SF12 and HLS_19_-Q12-NO, have been well studied and validated for the adult populations [21, 23, 31, 32], this study, to our knowledge, is the first one that simultaneously assessed the psychometric properties of all recently suggested 12-item versions of the HLS_19_-Q47 applied in YP aged 16–25.

Based on data from the Norwegian HLS_19_ study, the empirical evidence has weakened our null hypothesis associated with the psychometric properties of the previously 12-item short versions of the HLS_19_-Q47, i.e., HLS_19_-Q12, HLS_19_-SF12, and HLS_19_-Q12-NO. By examining poorly fitting items displayed from Rasch modelling and CFA, we successfully established a psychometrically sound parsimonious 12-item version (HLS_19_-YP12) for use among YP aged 16–25 years.

The empirical evidence suggested that the HLS_19_-YP12 has superior psychometric properties and convincingly outperforms other recently available 12-item short versions of the HLS_19_-Q47, i.e., HLS_19_-Q12, HLS_19_-SF12, and HLS_19_-Q12-NO.

### Psychometric properties of the 12-item versions; HLS_19_-YP12, HLS_19_-Q12-NO, HLS_19_-Q12, and HLS_19_-SF12 at the overall level

#### Dimensionality

Previous research has concluded that the HLS_19_-Q12-NO was psychometrically superior to other short versions of the HLS_19_-Q47 [21, 31]. However, the HLS_19_-Q12 was not reviewed in these studies. Nonetheless, all short versions have been suggested and validated for adult populations. Applied in data from YP, the HLS_19_-Q12-NO still seemed to fit the unidimensional Rasch model better than the other two scales, HLS_19_-Q12 and HLS_19_-SF12. Nevertheless, the present study provided evidence that the suggested HLS_19_-YP12 displayed even better fit to the unidimensional Rasch model than did the HLS_19_-Q12-NO, and unconditionally stood out as sufficiently unidimensional.

Applying the guidelines for CFA in Mplus set forth in Asparouhov and Muthén [63], established approximate fit was only tenable when SRMR ≤ 0.080 and all residuals were small (r_res_ ≤ 0.10). Asparouhov and Muthén [63] claim that it would be inaccurate to consider models that have large residual values as approximately well-fitting models, as large residual values indicate major discrepancy between the model and the data. However, we exceptionally considered it acceptable if only remarkable few residuals that were barely higher than 0.10. Disregarding some residuals higher than 0.10, other GOF indices, such as RMSEA, CFI, and TLI, indicated that both one- and three-factorial models of the HLS_19_-Q12 and the HLS_19_-Q12-NO have relatively good data-model fit. Furthermore, the HLS_19_-SF12 did also display acceptable data-model fit based on these GOF indices. Nevertheless, researchers have discussed whether it is expedient to assess the other GOF indices (RMSEA, CFI, and TLI) when the criterion of SRMR and all small residuals are not met [63]. Large residual values indicate that model modifications are needed.

Based on our national representative sample (*n* = 890) of youth aged 16–25 years, it is strongly evident that the one-factorial CFA model explains best the data from the HLS_19_-YP12 in comparison with other 12-item short versions, as well as the data from this scale fitted best the unidimensional polytomous Rasch model.

#### Targeting

All the 12-item short scales obtained a positive mean person location value indicating that the data as a whole was located at a higher level than the average of the scale. In other words, the items are deemed to be too easy for the participants’ ability. The HLS_19_-Q12 and the HLS_19_-SF12 obtained the highest values of mean person location, and we might have witnessed to the ceiling effect (extreme person scores), in which poor targeting have caused disordered response categories [69]. Out of the four 12-item short scales, the distribution of item-threshold locations and the distribution of person locations were best aligned for the HLS_19_-YP12 (Fig. 1), which is reflected by the lowest mean person location value (1.035). However, the instrument could benefit from adding items that are harder to endorse.

### Psychometric properties of the 12-item versions; HLS_19_-YP12, HLS_19_-Q12, HLS_19_-Q12-NO, and HLS_19_-SF12 at item level

#### Item fit

In accordance with results from the Rasch analyses of the HLS_19_-Q12 when applied in adult populations [32], item31 in the HLS_19_-Q12 also displayed poor item fit and was the only item within all four short versions that under-discriminated. In addition to item31, item28 deals with difficulties appraising and applying health information from “mass media” as there were added instructions guiding the participants to recognize what mass media (i.e., Newspapers, TV, or Internet) refers to [19]. The various types of media might have caused the undistinguished response pattern regardless of the participant’s HL level, as the difficulty of appraising or applying information from mass media might be dependent on what kind of media they refer to.

Applying the one-factorial CFA model (Table 6), item28 in the HLS_19_-Q12-NO displayed the second lowest factor loading while item31 in the HLS_19_-Q12 had the lowest loaded factor on their respective dimensions. Therefore, items referring to the mass media, likely perceived as digital resources, may be replaced by other items as they are more likely related to e.g., a digital HL construct, which is another aspect of the overall HL.

#### Differential item functioning

DIF for societal levels was observed for item14 […to follow instructions on medication] and item42 […to judge how your housing conditions may affect your health and well-being] in the HLS_19_-Q12-NO and the HLS_19_-Q12, respectively. Supplementary analyses were conducted to understand why DIF was displayed for societal level among YP. The results (not reported in the Tables) showed that while about 80% of the youngest subgroup placed themselves at the highest societal level, only 60% of the oldest subgroup did the same. It could be explained that different understandings of societal level among the youngest and the oldest subgroups, i.e., a 16-year-old might perceive not owing a popular piece of clothing, like an expensive jacket, as being placed at a very low level in the society, whereas a 25-year-old might have another opinion and perception based on the wider context. In turn, the different perceptions might have caused the DIF for societal levels observed in item14 and item42 in the HLS_19_-Q12-NO and the HLS_19_-Q12, respectively. However, there is no evidence of DIF for age groups.

Further investigation of reasons to why there is DIF for item14 and item42, a supplementary frequency analysis (not reported) was conducted showing that 89% of the youngest subgroup answered (very) easy on item14: to follow instructions on medication. Shed light on this result, one might recognize that parents could have played an important role giving YP both a reminder and guidance [70] concerning medications and applying the information provided from the doctor with regard to medications. Surprisingly, the same proportions (80%) of both age subgroups have answered (very) easy on item42, as one might have expected a higher proportion of the youngest who experienced it more difficult considering that they are still living at their parents’ place. This demonstrates that the YP are as reflected as the adult population in these kind of questions, yet this phenomenon is to be investigated further in more details.

#### Ordered response categories

Disordered response categories might be explained by too few persons located at the specific threshold levels and it is most likely due to bad targeting as well [71]. Item16 in the HLS_19_-Q12 showed that the first two thresholds were very close together and slightly reversed. More severely disordered response categories were identified on item15 in the HLS_19_-SF12, in which the two first thresholds were clearly reversed. The latter case weakened the hypothesis of ordinal data.

#### Content validity

Item13, item36, item41, and item46 in the HLS_19_-YP12 are the unique items and distinguished from the other three 12-item scales. The remaining eight items (item4, item7, item10, item18, item23, item26, item30 and item38) are to be found in either the HLS_19_-Q12, the HLS_19_-SF12, or the HLS_19_-Q12-NO. Especially item26 […to judge which vaccinations you or your family may need] and item36 […to find information about how to promote health at work, at school, or in the neighborhood] are particularly relevant to YP, as they still have to deal with, e.g., vaccination programs and other health related issues at school age. Adopting these two items in the new 12-item short version contributed to responding on the critique from Bröder et al. [72] concerning the lack of YP’s specific needs and social structures in most of the models.

However, the face validity has not been explicitly performed for the instruments beforehand towards participants aged 16–17 years. This age group and 18-year-old persons, most likely represent pupils in the upper secondary school, so that the readability and response burden for this group was assumed not critically derived from the burden separately applied to persons aged 18 years. When examining the response time median (range: 16.7—18.9 min), it is evident that the response burden was not different for the 16- and 17-year-old participants (17.7 and 17.3 min, respectively) compared to participants aged 18–25 (range: 16.7—18.9 min). Even though the understandability of item content has been ensured through cognitive interviews in young adults aged 18 and above, more interviews may be considered for YP below 18, confirming that the items are also well understood in this target group.

Notably, one of the strengths of the HLS_19_-YP12 instrument is that it was developed based on a definition and conceptual framework of HL, by which the content validity has been ensured. Furthermore, the new instrument has included items that are considered more likely related to younger people, such as vaccination and health promoting activities in school and neighborhood. As for the scale’s targeting, the distribution of both item-threshold and person locations were best aligned for the HLS_19_-YP12, indicating that the content in the new instrument was better adapted to the target population.

Finally, YP are expected to use social media and digital platforms actively to access health information [6, 73]. Surprisingly, items related to mass media, e.g., item28 in the HLS_19_-Q12-NO and item31 in the HLS_19_-Q12 tend to under-discriminate. A prior study [3] might have provided the explanation, that YP preferred to utilize their family as information resources rather than social media platforms. Furthermore, YP might have perceived mass media as part of another construct relative to digital health information platforms and skills.

### Limitations

The sample size of the HLS_19_-Q47, the HLS_19_-YP12, and the HLS_19_-SF12 was limited to n = 419. Therefore, all analyses that aimed to compare the various short versions were based on this sample size. There are no strict requirements for sample size in Rasch modelling. However, a rule of thumb assumes the useful sample size for a test of 12 polytomous items with 3 thresholds should comprise at least 360 up to 720 persons, in which a reasonable ratio is between 10 to 20 persons for each threshold [60]. Mundfrom et al. [74] suggested that the minimum sample size for applying CFA is depending on the variables-to-factors ratio and the number of factors that are present in the data. However, Hair et al. [75] claimed that a sample size above 300 are unlikely to produce Heywood cases. Hence, we assumed that our sample size of *n* = 419 was sufficient for the analyses performed. Taking into consideration that data-model fit and analysis of DIF in Rasch modelling and exact fit in CFA both are relatively sensitive to sample size, in which DIF in Rasch modelling is more likely with larger sample size and model Chi-square significance in CFA is more sensitive to smaller sample size. Thus, interpretation of the findings might be doing with some cautions.

In this study, we have applied both modern (Rasch modelling) and classical test theory (CFA). However, future research may also consider other relevant modern short-form development techniques. Finally, the HLS_19_-YP12 was developed and psychometrically assessed based on national data. Hence, the psychometric properties of the instruments should be further assessed using multinational data.

## Conclusions

The revised version of HLS-EU-Q47 (HLS_19_-Q47) was supplementarily confirmed to fit a 12-dimensional model best. Hence, it is not statistically defensible to report total score for individuals based on this scale as the person estimates of HL (person locations) cannot derive from her/his raw score from the multidimensional scale. This principle also applies to all short versions that are not sufficiently unidimensional.

Remaining as the best-fitted 12-item short version to the unidimensional Rasch model and the one-factorial CFA, including factor loading > 0.500 achievement for all items, the HLS_19_-YP12 is the first sufficiently unidimensional and conceptually developed HL instrument towards young people aged 16–25. This instrument is psychometrically superior and convincingly outperformed the other three 12-item short versions. Consequently, the HLS_19_-YP12 offers an efficient and much-needed screening tool for use among YP, which is likely a useful application in processes towards the development and evaluation of health policy and public health work, as well as for use in clinical settings.

Based on relatively strong evidence from the study, we suggest that the HLS_19_-YP12 instrument (Table S5) is preferred in future studies measuring HL among YP from the age of 16.

## Supplementary Information

**Additional file 1:** **Table S1.** Overall fit statistics applying unidimensional and multidimensional Rasch models of HLS_19_-Q47 and its short versions.

**Additional file 2:** **Table S2.** Entries in the residual correlation matrix for the 12-item short scales.

**Additional file 3:** **Table S3.** Fit statistics for different factor structures applying confirmatory factor analyses of the HLS_19_-Q47.

**Additional file 4:** **Table S4.** Item characteristics and DIF of HLS_19_-Q47 applying the 12-dimensional Rasch model.

**Additional file 5:** **Table S5.** The HLS_19_-YP12 instrument with response options.

## Data Availability

The datasets used and/or analyzed during the current study are not publicly available, but can be accessed by applying to the Norwegian Study Centre of HLS_19_ via this website: https://www.oslomet.no/forskning/forskningsprosjekter/befolkningens-helsekompetanse-hls19

## Abbreviations

A: Apply health information
AIC: Akaike’s information criterion
ANOVA: Two-way analysis of variance
CFA: Confirmatory factor analysis
CFI: Comparative fit index
CI: Confidence interval
df: Degree of freedom
DP: Disease prevention
DIF: Differential item functioning
ep: Estimated parameters
F: Finding health information
FIML: Information maximum likelihood
HC: Health care
HL: Health literacy
HLS-EU-Q47: European Health Literacy Survey Questionnaire
HLS_19_-Q47: European Health Literacy Population Survey 2019–2021 (HLS_19_) Questionnaire
HP: Health promotion
ICC: Item characteristic curve
IRT: Item response theory
J: Judging/appraising health information
MMLE: Marginal maximum likelihood estimation
MNSQ: Mean square
M-POHL: WHO action network on measuring population and organisational health literacy
MRCML model: “Multidimensional random coefficients multinomial logit” model
PCA: Principal component analysis
PMLE: Pairwise maximum likelihood estimation
PSI: Person separation index
PSR: Person separation reliability
RMSEA: Root-mean-squared error of approximation
SRMR: Standardized root mean square residual
SD: Standard deviation
TLI: Tucker and Lewis fit index
U: Understanding health information
WLE: Warm’s mean weighted likelihood estimation
WLSMV: Weighted least square mean and variance estimators
