# Supplementary material for: Developing the HLS19-YP12 for measuring health literacy in young people: a latent trait analysis using Rasch modelling and confirmatory factor analysis
Source: BMC Health Serv Res. 2022 Dec 6;22:1485. doi: 10.1186/s12913-022-08831-4 (PMC9727937; doi:10.1186/s12913-022-08831-4)
Supplement: Supplementary file 2 — Additional file 2: Table S2. Entries in the residual correlation matrix for the 12-item short scales. [file 12913_2022_8831_MOESM2_ESM.docx]

**Table S2:** Entries in the residual correlation matrix for the 12-item short scales

**Table S2a1**: Entries in the residual correlation matrix based on the HLS_19_-YP12, applying one-factor model

| Item relative to HLS_19_-Q47 | Item4 | Item7 | Item10 | Item13 | Item18 | Item23 | Item26 | Item30 | Item36 | Item38 | Item41 | Item46 |
| --- | --- | --- | --- | --- | --- | --- | --- | --- | --- | --- | --- | --- |
| Item4 |  |  |  |  |  |  |  |  |  |  |  |  |
| Item7 | .03 |  |  |  |  |  |  |  |  |  |  |  |
| Item10 | -.02 | -.01 |  |  |  |  |  |  |  |  |  |  |
| Item13 | .07 | -.03 | .06 |  |  |  |  |  |  |  |  |  |
| Item18 | -.00 | .06 | -.02 | -.02 |  |  |  |  |  |  |  |  |
| Item23 | -.03 | -.03 | -.03 | -.03 | .01 |  |  |  |  |  |  |  |
| Item26 | -.03 | .04 | .01 | -.02 | .00 | .03 |  |  |  |  |  |  |
| Item30 | .02 | .02 | -.08 | -.01 | -.01 | .05 | .09 |  |  |  |  |  |
| Item36 | -.06 | -.03 | .05 | -.03 | -.03 | -.05 | -.06 | -.01 |  |  |  |  |
| Item38 | -.02 | -.02 | .04 | .01 | .02 | .08 | -.07 | -.00 | .01 |  |  |  |
| Item41 | -.03 | -.04 | .02 | -.02 | .00 | .03 | -.03 | -.04 | .10 | -.10 |  |  |
| Item46 | .05 | -.01 | -.06 | .02 | .00 | -.04 | -.02 | -.05 | .08 | .00 | .02 |  |

**Table S2a2**: Entries in the residual correlation matrix based on the HLS_19_-YP12, applying three-factor model

| Item relative to HLS_19_-Q47 | Item4 | Item7 | Item10 | Item13 | Item18 | Item23 | Item26 | Item30 | Item36 | Item38 | Item41 | Item46 |
| --- | --- | --- | --- | --- | --- | --- | --- | --- | --- | --- | --- | --- |
| Item4 |  |  |  |  |  |  |  |  |  |  |  |  |
| Item7 | .02 |  |  |  |  |  |  |  |  |  |  |  |
| Item10 | -.04 | -.03 |  |  |  |  |  |  |  |  |  |  |
| Item13 | .06 | -.05 | .04 |  |  |  |  |  |  |  |  |  |
| Item18 | .00 | .06 | -.01 | -.02 |  |  |  |  |  |  |  |  |
| Item23 | -.02 | -.02 | -.02 | -.02 | -.02 |  |  |  |  |  |  |  |
| Item26 | -.02 | .05 | .02 | -.01 | -.03 | -.01 |  |  |  |  |  |  |
| Item30 | .03 | .03 | -.07 | -.00 | -.04 | .02 | .06 |  |  |  |  |  |
| Item36 | -.06 | -.02 | .05 | -.02 | -.02 | -.04 | -.04 | .00 |  |  |  |  |
| Item38 | -.02 | -.01 | .05 | .02 | .03 | .10 | -.06 | .01 | -.03 |  |  |  |
| Item41 | -.02 | -.03 | .03 | -.02 | .02 | .05 | -.01 | -.02 | .08 | **-.13** |  |  |
| Item46 | .06 | .00 | -.05 | .02 | .02 | -.02 | -.00 | -.03 | .05 | -.03 | -.01 |  |

Values < -.10 and > .10 are in bold.

**Table S2b1**: Entries in the residual correlation matrix based on the HLS_19_-Q12, applying one-factor model

| Item relative to HLS_19_-Q47 | Item4 | Item7 | Item10 | Item16 | Item18 | Item23 | Item24 | Item31 | Item32 | Item37 | Item42 | Item44 |
| --- | --- | --- | --- | --- | --- | --- | --- | --- | --- | --- | --- | --- |
| Item4 |  |  |  |  |  |  |  |  |  |  |  |  |
| Item7 | .01 |  |  |  |  |  |  |  |  |  |  |  |
| Item10 | -.00 | .06 |  |  |  |  |  |  |  |  |  |  |
| Item16 | .06 | -.02 | .04 |  |  |  |  |  |  |  |  |  |
| Item18 | -.04 | .06 | .02 | -.01 |  |  |  |  |  |  |  |  |
| Item23 | -.07 | -.01 | .03 | .06 | -.01 |  |  |  |  |  |  |  |
| Item24 | .01 | .02 | .02 | .04 | -.08 | .07 |  |  |  |  |  |  |
| Item31 | -.06 | .00 | -.01 | **-.11** | -.01 | -.04 | .00 |  |  |  |  |  |
| Item32 | .01 | -.05 | **-.18** | -.03 | .03 | -.02 | .07 | .01 |  |  |  |  |
| Item37 | -.01 | -.03 | -.04 | .00 | -.04 | -.00 | -.06 | **.11** | .09 |  |  |  |
| Item42 | .02 | -.04 | -.00 | -.10 | -.00 | .03 | -.03 | .01 | -.06 | -.04 |  |  |
| Item44 | .03 | -.02 | -.02 | -.04 | .06 | **-.11** | **-.11** | .03 | .04 | -.01 | **.14** |  |

*Note*: Values < -.10 and > .10 are in bold.

**Table S2b2**: Entries in the residual correlation matrix based on the HLS_19_-Q12, applying three-factor model

| Item relative to HLS_19_-Q47 | Item4 | Item7 | Item10 | Item16 | Item18 | Item23 | Item24 | Item31 | Item32 | Item37 | Item42 | Item44 |
| --- | --- | --- | --- | --- | --- | --- | --- | --- | --- | --- | --- | --- |
| Item4 |  |  |  |  |  |  |  |  |  |  |  |  |
| Item7 | -.01 |  |  |  |  |  |  |  |  |  |  |  |
| Item10 | -.03 | .04 |  |  |  |  |  |  |  |  |  |  |
| Item16 | .03 | -.05 | .02 |  |  |  |  |  |  |  |  |  |
| Item18 | -.05 | .06 | .01 | -.01 |  |  |  |  |  |  |  |  |
| Item23 | -.07 | -.01 | .02 | .05 | -.01 |  |  |  |  |  |  |  |
| Item24 | .01 | .01 | .01 | .03 | -.08 | .07 |  |  |  |  |  |  |
| Item31 | -.06 | -.00 | -.01 | **-.11** | -.01 | -.04 | .00 |  |  |  |  |  |
| Item32 | .04 | -.03 | **-.15** | -.01 | .04 | -.01 | .08 | .01 |  |  |  |  |
| Item37 | .02 | .00 | -.01 | .03 | -.04 | .00 | -.05 | **.12** | .05 |  |  |  |
| Item42 | .04 | -.01 | .02 | -.07 | .00 | .04 | -.03 | .01 | -.09 | -.07 |  |  |
| Item44 | .06 | .01 | .00 | -.01 | .06 | -.10 | -.10 | .03 | .00 | -.04 | .10 |  |

*Note*: Values < -.10 and > .10 are in bold.

**Table S2c1:** Entries in the residual correlation matrix based on the HLS_19_-SF12, applying one-factor model

| Item relative to HLS_19_-Q47 | Item2 | Item6 | Item10 | Item15 | Item18 | Item23 | Item26 | Item30 | Item33 | Item39 | Item43 | Item45 |
| --- | --- | --- | --- | --- | --- | --- | --- | --- | --- | --- | --- | --- |
| Item2 |  |  |  |  |  |  |  |  |  |  |  |  |
| Item6 | -.07 |  |  |  |  |  |  |  |  |  |  |  |
| Item10 | .00 | **.13** |  |  |  |  |  |  |  |  |  |  |
| Item15 | .03 | -.01 | -.08 |  |  |  |  |  |  |  |  |  |
| Item18 | .05 | -.10 | .03 | .01 |  |  |  |  |  |  |  |  |
| Item23 | -.05 | .05 | .03 | -.01 | -.01 |  |  |  |  |  |  |  |
| Item26 | .08 | .08 | .10 | -.05 | .01 | .03 |  |  |  |  |  |  |
| Item30 | -.03 | .06 | -.04 | -.07 | -.04 | .01 | .08 |  |  |  |  |  |
| Item33 | .01 | -.06 | **-.13** | .08 | .01 | **-.15** | **-.17** | -.05 |  |  |  |  |
| Item39 | -.07 | -.04 | -.10 | -.03 | .04 | .09 | **-.12** | .07 | .06 |  |  |  |
| Item43 | -.02 | -.03 | .06 | .01 | -.02 | -.01 | -.03 | -.09 | **.11** | -.02 |  |  |
| Item45 | .00 | -.08 | **-.12** | .09 | .00 | -.05 | **-.17** | .01 | **.17** | .02 | .06 |  |

*Note*: Values < -.10 and > .10 are in bold.

**Table S2c2:** Entries in the residual correlation matrix based on the HLS_19_-SF12, applying three-factor model

| Item relative to HLS_19_-Q47 | Item2 | Item6 | Item10 | Item15 | Item18 | Item23 | Item26 | Item30 | Item33 | Item39 | Item43 | Item45 |
| --- | --- | --- | --- | --- | --- | --- | --- | --- | --- | --- | --- | --- |
| Item2 |  |  |  |  |  |  |  |  |  |  |  |  |
| Item6 | -.08 |  |  |  |  |  |  |  |  |  |  |  |
| Item10 | -.01 | **.12** |  |  |  |  |  |  |  |  |  |  |
| Item15 | .03 | -.02 | -.09 |  |  |  |  |  |  |  |  |  |
| Item18 | .04 | **-.11** | .02 | -.01 |  |  |  |  |  |  |  |  |
| Item23 | -.06 | .03 | .01 | -.02 | -.03 |  |  |  |  |  |  |  |
| Item26 | .06 | .06 | .08 | -.07 | -.01 | .01 |  |  |  |  |  |  |
| Item30 | -.04 | .04 | -.06 | -.08 | -.05 | -.01 | .06 |  |  |  |  |  |
| Item33 | .02 | -.04 | **-.11** | **.11** | .04 | **-.12** | **-.14** | -.02 |  |  |  |  |
| Item39 | -.05 | -.01 | -.07 | .00 | .07 | **.13** | -.09 | .10 | .00 |  |  |  |
| Item43 | .00 | -.01 | .08 | .03 | .01 | .02 | .00 | -.06 | .04 | -.09 |  |  |
| Item45 | .02 | -.06 | -.10 | **.12** | .03 | -.02 | **-.14** | .04 | **.11** | -.04 | -.01 |  |

*Note*: Values < -.10 and > .10 are in bold.

**Table S2d1:** Entries in the residual correlation matrix based on the HLS_19_-Q12-NO, applying one-factor model

| Item relative to HLS_19_-Q47 | Item2 | Item7 | Item10 | Item14 | Item18 | Item23 | Item28 | Item30 | Item32 | Item38 | Item43 | Item44 |
| --- | --- | --- | --- | --- | --- | --- | --- | --- | --- | --- | --- | --- |
| Item2 |  |  |  |  |  |  |  |  |  |  |  |  |
| Item7 | -.00 |  |  |  |  |  |  |  |  |  |  |  |
| Item10 | .01 | .07 |  |  |  |  |  |  |  |  |  |  |
| Item14 | **-.12** | .01 | -.04 |  |  |  |  |  |  |  |  |  |
| Item18 | .05 | .05 | -.01 | -.03 |  |  |  |  |  |  |  |  |
| Item23 | -.02 | .01 | .02 | .03 | -.03 |  |  |  |  |  |  |  |
| Item28 | .07 | .02 | .04 | **-.11** | .06 | .05 |  |  |  |  |  |  |
| Item30 | .01 | .05 | -.03 | .04 | -.04 | .05 | .06 |  |  |  |  |  |
| Item32 | .03 | -.06 | **-.20** | -.00 | -.01 | -.04 | -.05 | .01 |  |  |  |  |
| Item38 | -.08 | -.03 | .05 | .10 | -.05 | .03 | .00 | -.04 | .06 |  |  |  |
| Item43 | -.00 | **-.15** | .03 | .02 | -.05 | -.01 | -.08 | -.07 | .08 | -.03 |  |  |
| Item44 | .02 | .00 | -.01 | -.04 | .05 | -.10 | -.08 | -.02 | .03 | -.06 | **.14** |  |

*Note*: Values < -.10 and > .10 are in bold.

**Table S2d2:** Entries in the residual correlation matrix based on the HLS_19_-Q12-NO, applying three-factor model

| Item relative to HLS_19_-Q47 | Item2 | Item7 | Item10 | Item14 | Item18 | Item23 | Item28 | Item30 | Item32 | Item38 | Item43 | Item44 |
| --- | --- | --- | --- | --- | --- | --- | --- | --- | --- | --- | --- | --- |
| Item2 |  |  |  |  |  |  |  |  |  |  |  |  |
| Item7 | -.00 |  |  |  |  |  |  |  |  |  |  |  |
| Item10 | .01 | .07 |  |  |  |  |  |  |  |  |  |  |
| Item14 | **-.12** | .01 | -.04 |  |  |  |  |  |  |  |  |  |
| Item18 | .05 | .04 | -.02 | -.05 |  |  |  |  |  |  |  |  |
| Item23 | -.03 | -.01 | .00 | .01 | -.05 |  |  |  |  |  |  |  |
| Item28 | .07 | .01 | .03 | **-.12** | .04 | .02 |  |  |  |  |  |  |
| Item30 | .00 | .04 | -.04 | .02 | -.06 | .02 | .04 |  |  |  |  |  |
| Item32 | .03 | -.04 | **-.18** | .01 | .03 | -.01 | -.02 | .03 |  |  |  |  |
| Item38 | -.07 | -.01 | .06 | **.12** | -.02 | .06 | .03 | -.01 | .03 |  |  |  |
| Item43 | .01 | **-.13** | .04 | .03 | -.02 | .02 | -.06 | -.04 | .04 | -.07 |  |  |
| Item44 | .03 | .01 | -.00 | -.02 | .08 | -.07 | -.06 | .01 | -.01 | -.10 | .10 |  |

*Note*: Values < -.10 and > .10 are in bold.
