# Supplementary material for: Developing the HLS19-YP12 for measuring health literacy in young people: a latent trait analysis using Rasch modelling and confirmatory factor analysis
Source: BMC Health Serv Res. 2022 Dec 6;22:1485. doi: 10.1186/s12913-022-08831-4 (PMC9727937; doi:10.1186/s12913-022-08831-4)
Supplement: Supplementary file 5 — Additional file 5: Table S5. The HLS19-YP12 instrument with response options. [file 12913_2022_8831_MOESM5_ESM.docx]

**Table S5**: The HLS_19_-YP12 instrument with response options

| **Health  domain** | **Cognitive domain** | **Item no. and relative to the HLS_19_-Q47** | **On a scale from very easy to very difficult, how easy would you say it is to…** | **Very  difficult** | **Difficult** | **Easy** | **Very easy** |
| --- | --- | --- | --- | --- | --- | --- | --- |
| Healthcare | F | 1: HL04 | ...find out where to get professional help when you are ill?  [Instructions: such as doctor, nurse, pharmacist, psychologist] | 1 | 2 | 3 | 4 |
|  | U | 2: HL07 | ...understand information about what to do in a medical emergency? | 1 | 2 | 3 | 4 |
|  | J | 3: HL10 | ...judge the advantages and disadvantages of different treatment options? | 1 | 2 | 3 | 4 |
|  | A | 4: HL13 | ...use information your doctor gives to you to make decisions about your illness? | 1 | 2 | 3 | 4 |
| Disease  prevention | F | 5: HL18 | ...find information on how to handle mental health problems?  [Instructions: stress, depression or anxiety] | 1 | 2 | 3 | 4 |
|  | U | 6: HL23 | ...understand information about recommended health screenings or examinations?  [Instructions: e.g. colorectal cancer screening, measuring blood pressure, blood sugar test] | 1 | 2 | 3 | 4 |
|  | J | 7: HL26 | ...judge which vaccinations, you or your family may need? | 1 | 2 | 3 | 4 |
|  | A | 8: HL30 | ...decide how you can protect yourself from illness using advice from family or friends? | 1 | 2 | 3 | 4 |
| Health  promotion | F | 9: HL36 | ...find information about how to promote health at work, at school or in the neighbourhood? | 1 | 2 | 3 | 4 |
|  | U | 10: HL38 | ...understand information on food packaging? | 1 | 2 | 3 | 4 |
|  | J | 11: HL41 | ...judge how your neighborhood may affect your health and well-being?  [Instructions: Your community, your neighbourhood] | 1 | 2 | 3 | 4 |
|  | A | 12: HL46 | ...influence your living conditions that affect your health and well-being?  [Instructions: Drinking and eating habits, exercise etc.] | 1 | 2 | 3 | 4 |

*Note*: F = Find; U = Understand; J = Judge/Appraise; A = Apply
